# Curcumin Attenuates on Carbon Tetrachloride-Induced Acute Liver Injury in Mice via Modulation of the Nrf2/HO-1 and TGF-β1/Smad3 Pathway

**DOI:** 10.3390/molecules23010215

**Published:** 2018-01-19

**Authors:** Xinyan Peng, Chongshan Dai, Quanwen Liu, Junke Li, Jingru Qiu

**Affiliations:** 1 College of Food Engineering, Ludong University, 186 Middle Hongqi Road, Yantai 264025, China; ldu_lqw@163.com (Q.L.); junjunke@163.com (J.L.); qjr1110@163.com (J.Q.); 2 College of Veterinary Medicine, China Agricultural University, 2 Yuanmingyuan West Road, Beijing 100193, China; daichongshan@cau.edu.cn

**Keywords:** curcumin, acute liver injury, Nrf2/HO-1 pathway, oxidative stress, TGF-β1/Smad3 pathway

## Abstract

This study aimed to investigate the protective effect of curcumin against carbon tetrachloride (CCl_4_)-induced acute liver injury in a mouse model, and to explain the underlying mechanism. Curcumin at doses of 50, 100 and 200 mg/kg/day were administered orally once daily for seven days prior to CCl_4_ exposure. At 24 h, curcumin-attenuated CCl_4_ induced elevated serum transaminase activities and histopathological damage in the mouse’s liver. Curcumin pre-treatment at 50, 100 and 200 mg/kg significantly ameliorated CCl_4_-induced oxidative stress, characterized by decreased malondialdehyde (MDA) formations, and increased superoxide dismutase (SOD), catalase (CAT) activities and glutathione (GSH) content, followed by a decrease in caspase-9 and -3 activities. Curcumin pre-treatment significantly decreased CCl_4_-induced inflammation. Furthermore, curcumin pre-treatment significantly down-regulated the expression of TGF-β1 and Smad3 mRNAs (both *p* < 0.01), and up-regulated the expression of nuclear-factor erythroid 2-related factor 2 (Nrf2) and HO-1 mRNA (both *p* < 0.01) in the liver. Inhibition of HO-1 attenuated the protective effect of curcumin on CCl_4_-induced acute liver injury. Given these outcomes, curcumin could protect against CCl_4_-induced acute liver injury by inhibiting oxidative stress and inflammation, which may partly involve the activation of Nrf2/HO-1 and inhibition of TGF-β1/Smad3 pathways.

## 1. Introduction

Liver disease is a global health problem [1] with acute liver injury associated, in particular, with high mortality rates [2]. The molecular processes underlying the pathogenesis of acute liver injury are known to involve a complex interplay of oxidative stress, apoptosis, inflammation, and necrosis [3,4]. It is well known that carbon tetrachloride (CCl_4_)-induced acute liver injury in a murine model is a classical system for investigating potential hepato-protective agents, owing to the similarity of its molecular mechanism with acute chemical liver injury in humans [5,6,7,8]. The bioactivation of CCl_4_ strongly depends on a specific cytochrome P450 2E1 from the microsomal compartment of the liver to produce a highly reactive trichloromethyl free radical, which then initiates lipid peroxidation and cellular damage. One of most important factors in the process of CCl_4_-induced acute liver injury is oxidative stress theory, which is also a potential target of drug treatment or development [8]. The inflammation response is also an important event for CCl_4_-induced acute liver injury [9]. Previous studies have demonstrated that CCl_4_ exposure could trigger the production of some inflammatory mediators, including tumor necrosis factor-alpha (TNF-α), inducible nitric oxide synthase (iNOS), and cyclooxygenase-2 (Cox-2), through nuclear factor-kappaB (*NF-κB*) activation in the liver of rats or mice [9,10,11,12].

Nuclear-factor erythroid 2-related factor 2 (Nrf2) is a bZip (basic leucine Zipper) transcription factor and a member of the CNC (cap “n” collar) family of transcription factors [13,14]. Nrf2 plays a critical role in regulating antioxidant genes by binding to antioxidant-response elements (AREs) [13,14]. Its activation has been regarded as an attractive strategy for the prevention and treatment of the oxidative damage related to many diseases, including acute and chronic liver injury [15,16,17]. In addition, the activation of Nrf2 can cause the expression of heme oxygenase-1 (HO-1) to inhibit the nuclear translocation of *NF-κB*, the “housekeeping genes” of the inflammatory response [18]. Transforming growth factor-beta1 (TGF-β1) predominantly transmits cell signaling through a downstream mediator protein, Smad3, to produce a concomitant extracellular matrix (ECM) [5]. A study by Niu et al. showed that TGF-β1/Smad3 signaling was activated in the process of CCl_4_-caused acute liver injury in mice, and the overexpression of Smad3 aggravated the acute liver injury by promoting inflammatory response and hepatocytes apoptosis [5]. A study by Oh et al. showed that sulforaphane could attenuate hepatic fibrosis in a mouse model by Nrf2-mediated inhibition of the TGF-β1/Smad3 pathway [19]. 

Curcumin (curry powder) is an active component in turmeric rhizomes (*Curcuma longa* Linn). It has been reported that curcumin has antioxidant, anti-inflammatory, anti-apoptotic and anti-bacterial functions [20,21,22]. The previous study showed that curcumin could directly activate Nrf2 expression, then attenuate quinocetone and furazolidone-induced liver toxicity and colistin-induced neurotoxicity in vitro [23,24,25]. Up until now, there has been no data reporting on the impact of curcumin on the expression of the TGF-β1/Smad3 pathway during CCl_4_-induced acute liver injury [26]. In the current study, we investigated the impact of curcumin pre-treatment on CCl_4_-induced acute liver injury in a mouse model and the Nrf2/HO-1 and TGF-β1/Smad3 pathways in the roles of curcumin’s protective effects.

## 2. Results

### 2.1. Curcumin Ameliorates CCl_4_-Induced Acute Liver Injury in Mice

**Figure 1:**
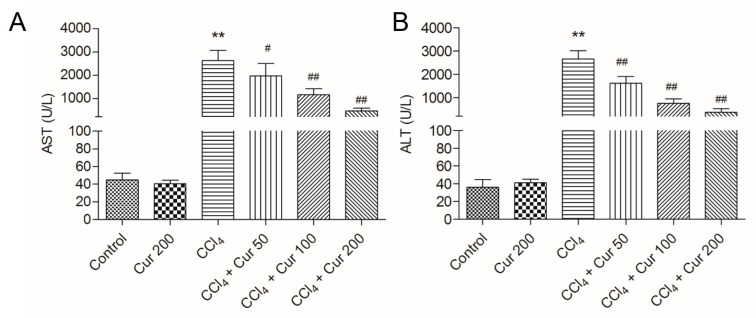
Effect of curcumin on the levels of aspartate aminotransferase (AST) and alanine transaminase (ALT). The levels of serum AST (**A**) and ALT (**B**) were examined at 24 h after CCl_4_ exposure. Data are presented as mean ± S.D. (*n* = 8 in each group). ** *p* < 0.01, compared with the control group; ^#^ *p* < 0.05 and ^##^ *p* < 0.01, compared with the CCl_4_ group.

First, we assessed the hepatoprotective effects of curcumin pre-treatment (at 50, 100 and 200 mg/kg/day for seven days) on CCl_4_-induced acute liver injury, while the levels of serum alanine transaminase (ALT) and aspartate aminotransferase (AST) and liver histology were considered as end points. As shown in Figure 1, serum AST and ALT activities in the CCl_4_ group significantly increased to 2623.2 U/L and 2655.8 U/L (both *p* < 0.01), compared to the control group. Curcumin pre-treatment at the doses of 50, 100 and 200 mg/kg (i.e., in the CCl_4_ + Cur 50, CCl_4_ + Cur 100 and CCl_4_ + Cur 200 groups, respectively) for seven days significantly decreased the levels of serum AST and ALT activities, compared to the CCl_4_ alone group.

**Figure 2:**
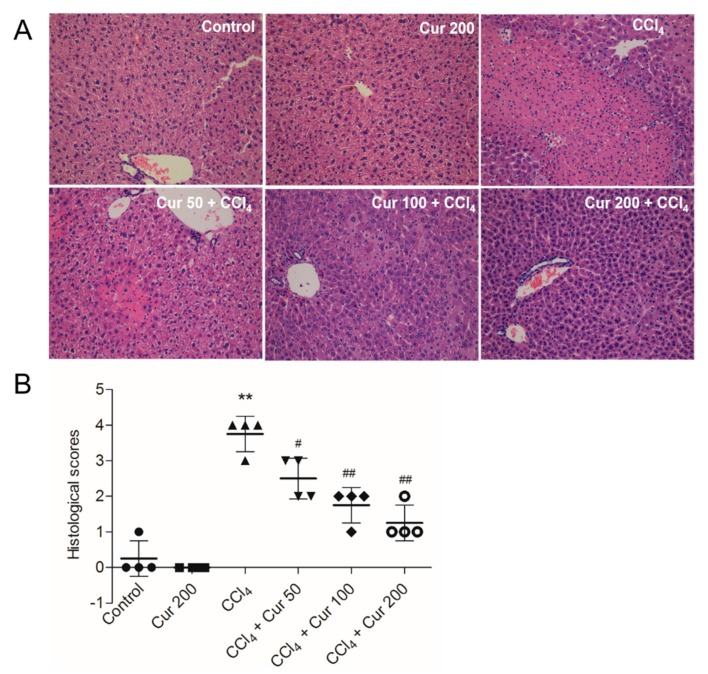
Effects of curcumin on CCl_4_-induced histopathological alterations. (**A**) Representative histopathological images of hmatoxylin and eosin (H&E) stained liver sections from control, curcumin, CCl_4_ and CCl_4_ plus curcumin treated mice, magnification 20×; (**B**) the histological scores for liver sections (*n* = 4). Data are presented as mean ± S.D. ** *p* < 0.01, compared to the control group; ^#^ *p* < 0.05 and ^##^ *p* < 0.01, compared to the CCl_4_ group.

At 24 h after CCl_4_ exposure, severe liver injury was evident, seen as large areas of extensive cellular necrosis with loss of hepatic architecture and inflammatory cell infiltration around the blood vessels (Figure 2A); consistently, the histological scores increased to 3.75 (*p* < 0.01) (Figure 2) compared to the control group. Curcumin pre-treatment attenuates CCl_4_-induced necrosis and inflammatory cell infiltration; correspondingly, histological scores were significantly decreased to 2.5, 1.75 and 1.25 (all *p* < 0.05 or 0.01) in the CCl_4_ + Cur 50, CCl_4_ + Cur 100 and CCl_4_ + Cur 200 groups, respectively (Figure 2B). Compared to the control group, there were no abnormal histological changes in the livers of mice in the solely curcumin group.

### 2.2. Curcumin Ameliorates CCl_4_-Induced Oxidative Stress in Liver Tissue

As shown in Table 1, CCl_4_ exposure for 24 h significantly increased the levels of malondialdehyde (MDA) to 2.79 mmol/mg protein, decreased the activities of superoxide dismutase (SOD), catalase (CAT) and levels of glutathione (GSH) to 78.4 U/mg protein, 86.5 U/mg protein, 38.2 mmol/mg protein (all *p* < 0.01), respectively. Curcumin pre-treatment, particularly at 100 mg/kg/day and 200 mg/kg/day, significantly attenuated all of these CCl_4_-induced biomarkers of oxidative stress (Table 1). Curcumin alone treatment at 200 mg/kg/day for seven days did not affect the changes of levels of MDA and GSH and the activities of SOD and CAT, compared to the control group (Table 1).

### 2.3. Curcumin Ameliorates CCl_4_-Induced Inflammatory Response in Livers of Mice

**Figure 3:**
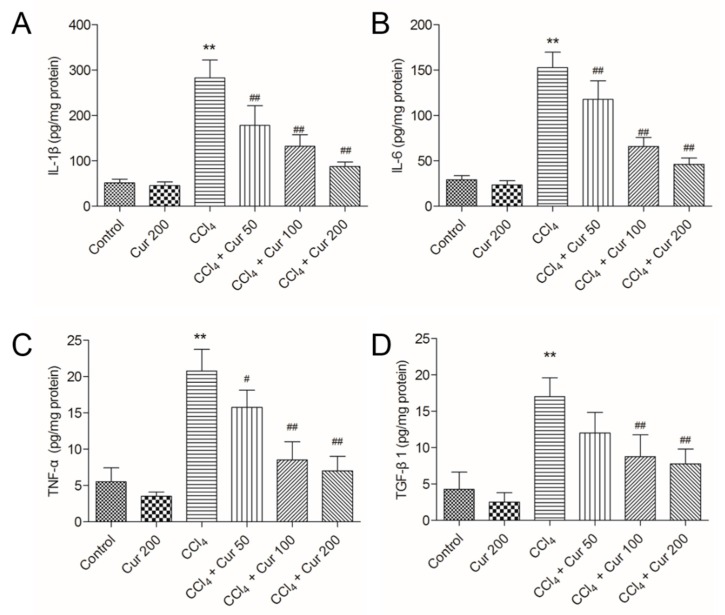
Effect of curcumin on the levels of interleukin (IL)-1β, IL-6, tumor necrosis factor (TNF)-α, and Transforming growth factor-beta1 (TGF-β1) proteins. The levels of IL-1β (**A**), IL-6 (**B**), TNF-α (**C**) and TGF-β1 (**D**) proteins were examined by using the enzyme-linked immunosorbent assay (ELISA) method. Results are presented as the mean ± S.D. (*n* = 8 in each group). ** *p* < 0.01, compared to the control group; ^#^ *p* < 0.05 and ^##^ *p* < 0.01 compared to the solely CCl_4_ treatment group.

As shown in Figure 3, CCl_4_ treatment significantly increased the expression levels of IL-1β, IL-6, TNF-α and TGF-β1 proteins to 282.6 pg/mg protein, 152.5 pg/mg protein, 20.8 pg/mg protein and 17.4 pg/mg protein (all *p* < 0.01), respectively, compared to the control group. Curcumin pre-treatment markedly attenuated the levels of IL-1β, IL-6, TNF-α and TGF-β1 in a dose-dependent manner (Figure 3). 

### 2.4. Curcumin Down-Regulates the Activities of Caspase-9 and -3 

**Figure 4:**
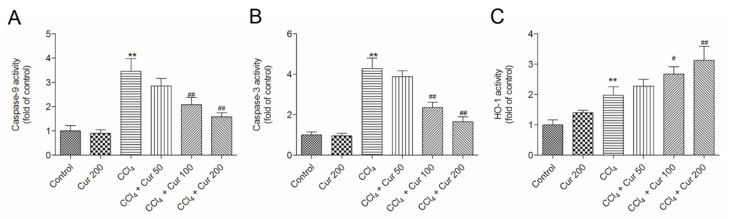
Effect of curcumin on the activities of caspase-9, -3 and HO-1. (**A**), caspase-9 activity; (**B**), caspase-3 activity; (**C**), HO-1 activity. Data are presented as mean ± S.D. (*n* = 8). ** *p* < 0.01, compared to the control group; ^#^ *p* < 0.05 and ^##^ *p* < 0.01, compared to the solely CCl_4_ treatment group.

Compared to the control, CCl_4_ treatment significantly increased the activities of caspase-9 and -3 to 3.5- and 4.3-fold (both *p* < 0.01), respectively (Figure 4). Curcumin pre-treatment markedly attenuated CCl_4_-induced increases of caspase-9 and -3 activities (Figure 4A,B) in a dose-dependent manner. In the CCl_4_ + Cur 100 and CCl_4_ + Cur 200 groups, caspase-9 activities decreased 2.1-fold and 1.6-fold, and caspase-3 activities decreased 2.4-fold and 1.7-fold (all *p* < 0.01), respectively, compared to the solely CCl_4_ treatment group. Meanwhile, compared to the control, CCl_4_ treatment markedly increased HO-1 activity, which was further increased 2.7-fold and 3.2-fold in the CCl_4_ + Cur 100 and CCl_4_ + Cur 200 groups (Figure 4C). There was no marked change in the activities of caspase-9 and -3 in the curcumin 200 mg/kg group, compared to the control group (Figure 4A,B). HO-1 activity was higher in all of the curcumin-treated groups than in the control group (Figure 4C).

### 2.5. Curcumin Up-Regulates Nrf2 Expression and Down-Regulates the Expressions of TGF-β1 and Smad3 Proteins

**Figure 5:**
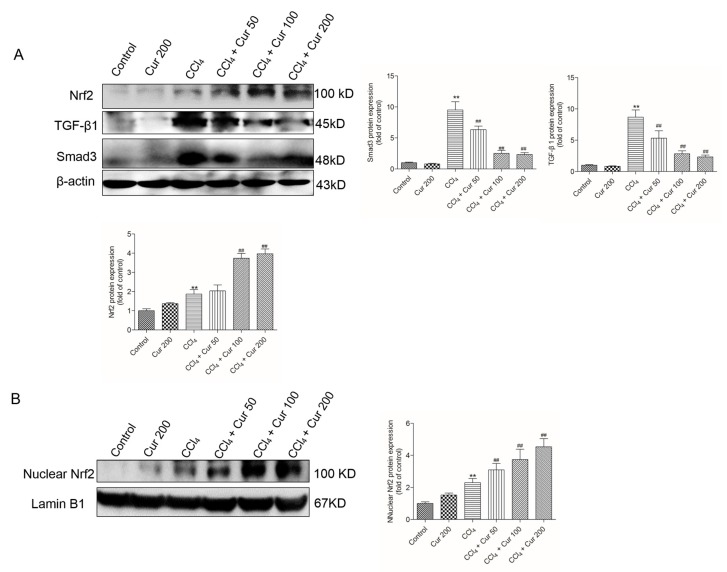
Effect of curcumin on the CCl_4_ treatment-induced expressions of Nrf2, TGF-β1 and Smad3 proteins. (**A**) Nrf2 (total), TGF-β1 and Smad3 protein expression were examined using the western blot and analyzed using Image J software. (**B**) Nuclear Nrf2 protein expression was examined using the western blot and analyzed using Image J software. Data are shown as mean ± S.D. (*n* = 3). ** *p* < 0.01, compared to the control group; ^##^ *p* < 0.01, compared to the solely CCl_4_ treatment group.

At 24 h, CCl_4_ treatment significantly up-regulated the protein expressions of total Nrf2 (increased 1.9-fold), TGF-β1 (increased 8.7-fold) and Smad3 proteins (9.5-fold) (all *p* < 0.01), compared to the control group. In the CCl_4_ + Cur 100 and CCl_4_ + Cur 200 groups, total Nrf2 protein expression increased 3.6 and 4.0-fold (both *p* < 0.01), respectively; TGF-β1 protein expression decreased 2.9-fold and 2.4-fold (both *p* < 0.01), respectively; Smad3 protein expression decreased 2.5-fold and 2.3-fold (both *p* < 0.01) (Figure 5A), respectively, compared to the solely CCl_4_ treatment group. Furthermore, Curcumin pre-treatment markedly promoted Nrf2 nuclear accumulation caused by CCl4 exposure (Figure 5B). Curcumin treatment also up-regulated the expression of Nrf2 and did not change the expression of the TGF-β1 and Smad3 proteins (Figure 5).

### 2.6. Curcumin Down-Regulates the Expressions of TGF-β and Smad3 mRNAs and Up-Regulates the Expressions of Nrf2 and HO-1 mRNAs 

**Figure 6:**
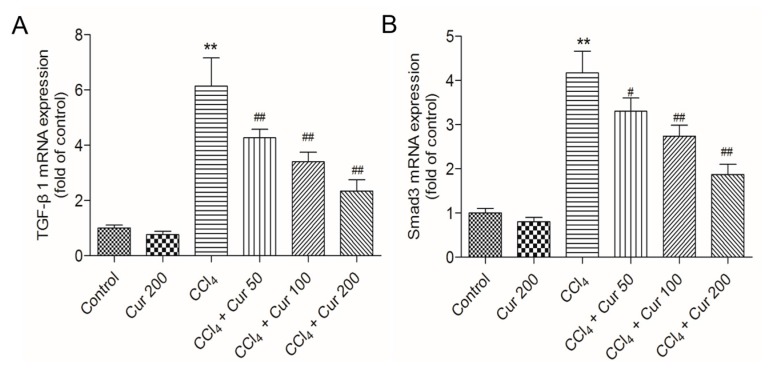

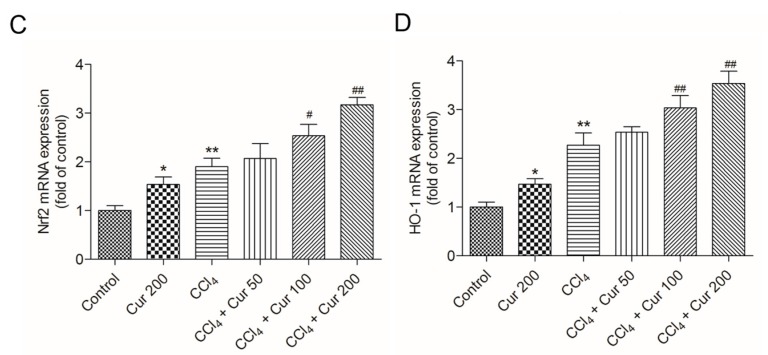
Effect of curcumin on CCl_4_-induced expression of TGF-β1 (**A**), Smad3 (**B**), Nrf2 (**C**) and HO-1 (**D**) mRNAs. Data are presented as mean ± S.D. (*n* = 8). *p < 0.05, ** *p* < 0.01, compared to the control group; ^#^ *p* < 0.05 and ^##^ *p* < 0.01, compared to the CCl_4_ group.

Compared to the control group, CCl_4_ treatment significantly up-regulated the expressions of TGF-β1 and Smad3 mRNAs 6.2-fold and 4.2-fold (both *p* < 0.01), respectively; it up-regulated the expressions of Nrf2 and HO-1 mRNAs 1.9-fold and 1.7-fold (both *p* < 0.01) (Figure 6). Curcumin pre-treatment, especially at 100 mg/kg/day and 200 mg/kg/day, significantly down-regulated the expressions of TGF-β1 and Smad3 mRNAs and up-regulated the expressions of Nrf2 and HO-1 mRNAs (all *p* < 0.05 or 0.01), compared to that in the solely CCl_4_ treatment group. Solely curcumin treatment at 200 mg/kg did not affect the expression of TGF-β1 and Smad3 mRNAs, but markedly up-regulated the expression of Nrf2 and HO-1 mRNAs (both *p* < 0.05) (Figure 6).

### 2.7. Inhibition of HO-1 Abolished the Protective Effect of Curcumin on CCl_4_-Induced Liver Injury

**Figure 7:**
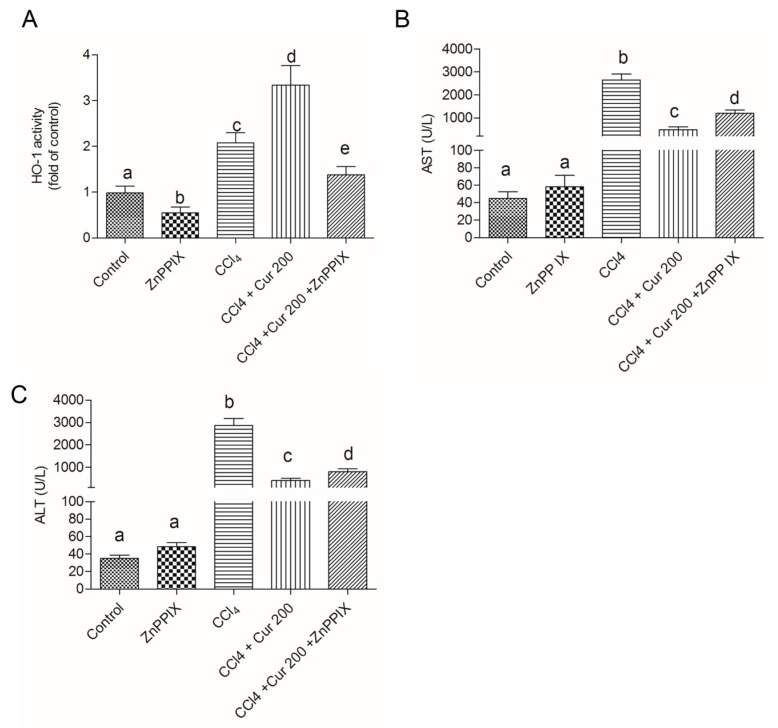
Inhibition of HO-1 abolished the protective effect of curcumin on CCl_4_-induced liver injury. (**A**) HO-1 activity and levels of serum AST (**B**) and ALT (**C**) were examined at 24 h after CCl_4,_ exposure. Data are presented as mean ± S.D. (*n* = 8 in each group). The values sharing the different letters are significantly different at *p* < 0.05 by Duncan’s multiple-range test.

As shown in Figure 7, HO-1 inhibitor ZnPP IX markedly inhibited the activity of HO-1 caused by curcumin (Figure 7A). Compared to the untreated group, significant increases of ALT and AST activities were detected in the CCl_4_, Cur 200 + CCl_4_, and Cur 200 + CCl_4_ + ZnPP IX groups. Compared to the Cur 200 + CCl_4_ group, HO-1 inhibition by ZnPPIX, i.e., in the Cur 200 + CCl_4_ + ZnPP IX group, significant increases of AST and ALT activity were detected (Figure 7B,C). Solely ZnPP IX treatment did not change the AST and ALT activity, compared to untreated mice in the control group. 

## 3. Discussion

CCl_4_ is a well-known hepatotoxin and CCl_4_-caused acute liver injury involves various mechanisms, including inflammatory responses, oxidative stress and apoptosis [6,10,27]. CCl_4_-induced acute liver injury in a murine model has usually been used for screening the anti-hepatotoxic and/or hepatoprotective activities of drugs [28]. Curcumin, a natural polyphenol in the spice turmeric, has many biological functions, such as anti-inflammatory, anti-oxidative, anti-carcinogenic and immuno-regulatory abilities [15]. Many studies have demonstrated that curcumin could protect against acute or chronic liver/kidney injury caused by drugs or toxins by scavenging reactive oxygen species (ROS) and improving anti-oxidative ability [29,30,31,32]. In the present study, curcumin pre-treatment exhibited a distinct protection against CCl_4_-induced acute liver injury, which was demonstrated by the decrease of serum ALT and AST levels and improved histological lesions (Figure 1 and Figure 2).

Lipid peroxidation was considered as one of the principal causes of CCl_4_-induced acute liver injury [33,34]. Antioxidant defense systems, including non-ezymatic antioxidants (e.g., GSH, vitamins C and E) and enzymatic activities such as SOD, CAT and HO-1, play a vital role in preventing damage caused by CCl_4_ metabolism driving oxygen-free radical production in the liver [7,10,11]. Curcumin pre-treatment at the doses of 50, 100 and 200 mg/kg markedly attenuated the increases of MDA level caused by CCl_4_ (Table 1). MDA is used as a biomarker of oxidative stress to evaluate the degree of the peroxidation of membrane lipids [35]. It indicated that curcumin could improve CCl_4_-induced lipid peroxidation. Studies have demonstrated that curcumin can directly interact with superoxide anion (O_2_^•−^) and hydrogen peroxide (H_2_O_2_) and inhibit oxidative stress even more than that of vitamin E, which is an oxygen-radical scavenger [35]. The previous study has demonstrated that curcumin can up-regulate the activities of SOD, CAT and levels of GSH, protects against glycerol-induced nephrotoxicity or diethylnitrosamine (DEN) induced hepatocarcinogenesis in a murine model [36]. Consistently, in the present study, solely curcumin treatment did not change the activity of SOD, CAT and levels of GSH, but markedly attenuated the decrease of these cellular antioxidants caused by CCl_4_ exposure (Table 1). In addition, the previous study showed that CYP2E1 is the greatest metabolism mediator of CCl_4_-generated reactive free radicals and curcumin could not moderate the decrease of CYP2E1 activity caused by CCl_4_ [37]. Consistently, these data indicated that that the protective role of curcumin against CCl_4_-induced oxidative stress may be attributed directly to scavenging O_2_^•−^ and H_2_O_2_. 

Necrosis and the apoptosis of hepatocytes are attributed to cell death in the centrilobular area during CCl_4_-induced acute liver injury [38]. Consistently, the current study showed that CCl_4_ treatment destroyed the normal cellular structure and resulted in the loss of nuclei and amounts of fragmental and condensed nucleus in a mouse’s liver tissue (Figure 2). Caspase-3 is a key biomarker of apoptosis [39]. Caspase 9 is an important biomarker in mitochondrial apoptosis and its activation could switch on caspase-3 mediated apoptosis [39]. A previous study showed that curcumin could inhibit oxidative stress and mitochondrial dysfunction-mediated caspase-3 activation, and attenuate galactosamine/lipopolysaccharide-induced acute liver injury in mice [33]. In the present study, curcumin pre-treatment markedly attenuated the pathology injury, as well as the increases of caspase-9 and -3 activity (Figure 4B,C). Similarly, the study of Xie et al. showed that CCl_4_ acute exposure-induced caspase activation-mediated apoptosis could be attenuated by the pre-treatment of dihydromyricetin, a natural compound [40]. Given this, it is very likely that curcumin could minimize CCl_4_-induced oxidative stress and the subsequent mitochondrial dysfunction and apoptosis.

It is well known that *NF-κB* activation can regulate the expression of over 500 genes associated with inflammation, tumorgenesis, cellular survival/proliferation, and chemoresistance [41]. Curcumin has a great deal of therapeutic potential for the treatment of inflammatory diseases due to its ability to inhibit *NF-κB* activation and its downstream genes including IL-1β, TNF-α and IL-6 expression [20,21,22]. In the present study, CCl_4_ treatment significantly increased the expression of IL-1β, TNF-α and IL-6 in mice liver tissues, which were effectively attenuated by curcumin pre-treatment in a dose-dependent manner (Figure 3). These data indicated that the anti-inflammation ability of curcumin may partly contribute to its protection against CCl_4_-induced acute liver injury in mice. Recently, a study by Bansal et al. showed that IL-6 might be hepato-protective in acute injury through down-regulation of MMP-2, an important factor in acute liver injury [42]. It has been demonstrated that inhibition of MMP-2 promoted acute liver injury, although the previous mechanism is still unclear [43]. Several studies also indicated that MMP2 could be down-regulated via the inhibition of *NF-κB* [44]. Therefore, the precise mechanism of IL-6 in the role of curcumin’s protection needs further investigation.

TGF-β1, a powerful profibrogenic cytokine, is a key mediator of hepatic stellate-cell activation [45]. The activation of TGF-β1 could cause accumulation of the extracellular matrix (ECM) proteins via the regulation of Smad3 that leads to fibrosis progression [5]. Recent studies have suggested that the activation of the TGF-β1/Smad3 pathway was associated with CCl_4_-induced acute liver injury; overexpression of TGF-β1 or Smad3 promoted a CCl_4_-induced inflammatory response and apoptosis in the hepatocyte of a mouse [5]. Consistent with this, the present study implies that CCl_4_ exposure markedly increases expression of TGF-β1 or Smad3 proteins and enhances the levels of mRNAs in liver tissue, which are markedly attenuated by curcumin pre-treatment (Figure 4, Figure 5 and Figure 6). Data indicated that TGF-β1/Smad3 may be a potential target of curcumin on protection against CCl_4_ induced acute liver injury. Similarly, a previous study has shown that curcumin pre-treatment could protect against sepsis-induced acute lung injury in a rat model by inhibiting the expression of the TGF-β1/Smad3 pathway [26].

Nrf2 is a transcription factor that induces expression of various cyto-protective enzymes including HO-1, and the activation of Nrf2 signaling is important for inhibiting oxidative stress and maintaining cellular homeostasis [13]. Recent studies have demonstrated that hepato-toxins such as bromobenzene, furosemide and CCl_4_ are capable of inducing a significant increase in Nrf2 accumulation in a liver injury [46]. In this study, CCl_4_ treatment significantly increased the expression of nuclear Nrf2 (Figure 5) and the expression of Nrf2 and HO-1mRNAs (Figure 6C,D), as well as HO-1 activity, which is consistent with previous studies from Su et al. and Choi et al. [47,48]. However, controversial results have also been reported, i.e., significant decreases of Nrf2 and HO-1 protein and mRNA expression have been detected after acute CCl_4_ exposure [49,50]. These differences may be related to the animal stains, time and dose of CCl_4_ exposure. It is widely accepted that antioxidants up-regulate the expressions of these protective genes although the effect of CCl_4_ is disputable. Growing evidence indicates that natural antioxidants play important roles in the activation of Nrf2 and HO-1, and many studies have demonstrated that curcumin is an activator of Nrf2 pathways [51]. Of particular interest, Nrf2 and HO-1 mRNAs expression and HO-1 activities further increased in the curcumin-treated CCl_4_ group (Figure 4C and Figure 6C,D), which implies a molecular basis by which curcumin stimulates the activity of antioxidants and phase II detoxifying enzymes. It has been reported that curcumin could trigger the Nrf2-mediated antioxidant response element (ARE) and induce some gene expression, including SOD and CAT or up-regulated GSH levels, when the cells are under oxidative stress conditions [35]. Interestingly, curcumin treatment activated Nrf2 and its down-stream gene HO-1 expression (Figure 5 and Figure 6), protecting against CCl_4_-induced oxidative stress. The further increase of Nrf2 and HO-1 in the curcumin plus CCl_4_ group (Figure 5 and Figure 6) may be also partly attributable to the feedback of the suitable recovery of the ROS. In addition, the inhibition of HO-1 partly attenuated the protective effect of curcumin against CCl_4_-induced acute liver injury (Figure 7). Taken together, the activation of Nrf2 may partly contribute to the protective role of curcumin against CCl_4_-induced acute liver injury. Some studies using a rat model have shown that Nrf2 mediated the inhibition TGF-β1/Smad3, contributing to the anti-fibrosis ability of curcumin [52]. This indicated that the activation of Nrf2 may partly contribute to the inhibition of curcumin on TGF-β1/Smad3 in liver tissue. 

In conclusion, our findings indicate that up-regulation of the Nrf2/HO-1 pathway and down-regulation of the TGF-β1/Smad3 pathway might be involved in the hepatoprotective effect of curcumin against CCl_4_-induced acute liver injury via the inhibition of oxidative stress and inflammatory response. The current study thus provides further insights into the protective effects of curcumin. The clinical relevance of these findings will have to be investigated in further studies

## 4. Materials and Methods

### 4.1. Chemicals

CCl_4_ was obtained from Kaixing Chemical Industry Co., Ltd. (Tianjin, China). Curcumin was purchased from Aladdin Reagent Co., Ltd. (Shanghai, China). Hemin, glucose-6-phosphate, and glucose-6-phosphate dehydrogenase were from Sigma-Aldrich (St. Louis, MO, USA). ZnPP was purchased from Sigma-Aldrich (Stockholm, Sweden). All other chemicals were of the highest analytical grade available.

### 4.2. Animal Treatment

All animal experiments were approved by the Institutional Animal Care and Use Committee at the China Agricultural University (ethical number CAU20170302-2). All animals were fed in the laboratory, which was maintained at approximately 22 °C and 50% relative humidity with a 12-h light–dark cycle. An acclimation period of 1 week was employed prior to the experiments. Mice had free access to food and water during the experiments.

Adult C57BL/6 mice (male, 6–8 weeks, 18–22 g) were purchased from Vital River Animal Technology Co., Ltd. (Beijing, China). Forty-eight mice were randomly divided in the following groups (*n* = 8 in each group): control, curcumin 200 mg/kg/day (curcumin 200 group); 0.5% CCl_4_ (CCl_4_ group); 0.5% CCl_4_ plus curcumin 50 mg/kg/day (CCl_4_ + curcumin 50 group); 0.5% CCl_4_ plus curcumin 100 mg/kg/day (CCl_4_ + curcumin 100 group); and 0.5% CCl_4_ plus curcumin 200 mg/kg/day (CCl_4_ + curcumin 200 group). In the CCl_4_ group, mice were intraperitoneally (i.p.) injected with 0.5% (*v*/*v*) CCl_4_ (10 mL/kg, dissolved in olive oil). In the CCl_4_ + curcumin 50, 100 and 200 groups, curcumin was suspended in 0.5% carboxyl methyl cellulose sodium (CMC-Na) and the doses of 50, 100 and 200 mg/kg were given orally for 7 days, and 0.5% CCl_4_ were i.p. injected at 2 h after the last dose of curcumin. The mice in the control and curcumin 200 groups were administrated with an equal volume of vehicle (oil + 0.5% CMC-Na) or curcumin (200 mg/kg). At 24 h after CCl_4_ injection, mice were sacrificed, and blood and liver samples were collected. One part of the liver was immediately fixed in 10% formaldehyde for histopathology observation and the rest was frozen with liquid nitrogen and stored at −80 °C until its use for biochemical, histopathological, cytokine and gene expression examinations, respectively.

To examine the role of the Nrf2/HO-1 pathway on the protective effect of curcumin against CCl_4_-induced acute liver injury, mice were divided into five groups (*n* = 8 in each group): control, zinc protoporphyrin IX (ZnPP) group (10 mg/kg); 0.5% CCl_4_ (CCl_4_ group); 0.5% CCl_4_ plus curcumin 200 mg/kg/day (CCl_4_ + Curcumin 200 group); and 0.5% CCl_4_ plus curcumin 200 mg/kg/day plus ZnPP IX group (CCl_4_ + Curcumin 200 + ZnPP IX group). Mice were pre-treated with ZnPP IX at 10 mg/kg at 12 h and 2 h before the last dose of curcumin administration, according to the previous study [53]. At 24 h after CCl_4_ injection, mice were sacrificed, and blood and liver samples were collected for measurement.

### 4.3. Measurement of Serum Alanine Transaminase (ALT) and Aspartate Transaminase (AST)

Blood samples were centrifuged at 3000× *g* for 10 min and the serum was collected. The levels of serum ALT and AST were determined by using an Automated Chemical Analyzer (Hitachi 7080, Hitachi High-Technologies Corporation, Shanghai, China) with the standard diagnostic kits (Shanghai Kehua Bio-Engineering Co., Ltd., Shanghai, China).

### 4.4. Histological Examination of Liver Damage

Parts of livers from each mouse (*n* = 4) were fixed in 10% neutral-buffered formalin for 48 h. The samples were de-waxed in xylene and rehydrated in a series of graded alcohols and then embedded in paraffin. The samples were sectioned at 4 μm and stained with hematoxylin-eosin (H.E.) for light microscopic examination. To examine the degree of necrosis of liver tissues, an injury grading score (Grade 0–4) based on severity of necrotic lesions in the liver parenchyma was carried out according to the previous study [54]. The scoring system was as follows: Grade 0, no pathological change; Grade 1, presence of degenerated hepatocytes with only rare foci of necrosis; Grade 2, small area of mild centrilobular necrosis around the central vein; Grade 3, area of mild centrilobular necrosis severer than Grade 2; and Grade 4, centrilobular necrosis severer than Grade 3. Twenty images were randomly selected from each slide of the sample and scored by two independent pathologists, and the values were analyzed.

### 4.5. Measurement of Activities of Superoxide Dismutase (SOD), Catalase (CAT) and Levels of Malondialdehyde (MDA) and Glutathione (GSH)

Liver tissues were homogenized at 4 °C in 9 volumes (approximately 1 mL per 0.1 g of tissue) of cold Tris buffer (0.01 M Tris-HCl, 0.1 mM EDTA-Na_2_, 0.01 M sucrose, 0.9% saline; pH 7.4). The resultant homogenates were centrifuged (14,000× *g*, 15 min) at 4 °C, and the supernatant was collected for assay of the levels of MDA and GSH and the activities of SOD and CAT by using commercial assay kits (Nanjing Jiancheng Bio-Corporation, Nanjing, China). The protein content was measured by using a bicinchoninic acid assay (BCA) kit (Nanjing Jiancheng Bio-Corporation, Nanjing, China).

### 4.6. Measurement of Caspase-9, -3 and HO-1 Activities

Liver-tissue samples were lysed using lysis buffer (1:10 *w*/*v*) for 15 min on ice, then centrifuged at 14,000× *g* for 15 min at 4 °C. The isolated supernatants were used to determine the activities of caspase-3 and caspase-9 (Beyotime, Haimen, China) using commercial enzyme-linked immunosorbent assay (ELISA) kits according to the manufacturer’s instructions, respectively. Total HO-1 activity was measured as described previously [55]. In brief, a 1.2-mL reaction system consists of 0.5 mg protein from liver-tissue homogenate, 2 mM glucose-6-phosphate, 0.2 U glucose-6-phosphate dehydrogenase, 0.8 mM NADP, and 20.0 mM hemin. The reaction system was incubated for 1 h at 37 °C, then its optical density at 464 nm against a baseline absorbance at 530 nm was determined using a multimode plate reader (Thermo Fisher Scientific, Waltham, MA, USA). The values in the different groups were normalized to the control for statistical analysis.

### 4.7. Measurement of TNF-α, IL-1β, IL-6 and TGF-β1 Levels

The levels of TNF-α, IL-1β, IL-6 and TGF-β1 in the liver tissues were measured using ELISA kits according to the manufacturer’s instructions (R&D Systems, Minneapolis, MN, USA). The levels were normalized to the value for the control.

### 4.8. Western Blotting

Western blotting was carried out according to the previous study with minor revision. In brief, mice liver tissues were lysed using an ice-cold lysis buffer (100 mM Tris-HCl, 2% (*w*/*v*) SDS, 10% (*v*/*v*) glycerol, pH 7.4); protease inhibitor cocktail (1 mM PMSF, 1 μg/mL aprotinin, 1 μg/mL leupeptin, and 1 μg/mL pepstatin A) was added to the lysis buffer before treatment. The samples were ultrasonicated (5 s ultrasonication and 6 s pause in each cycle for ×5, power 30 W) using an Ultrasonic Processor (Branson, MO, USA). The tissue lysates were centrifuged at 14,000× *g* for 15 min at 4 °C, and the supernatants were collected. NE-PER (Pierce Biotechnology, Rockford, IL, USA) was used for the extraction of nuclear proteins, according to the manufacturer’s instructions. The protein concentration was measured using the BCA protein assay kit. Equal amounts of protein from each sample were resolved by sodium dodecyl sulfate polyacrylamide gel electrophoresis (SDS-PAGE) and transferred to nitrocellulose membranes (Bio-Rad, Hemel Hempstead, UK). Primary rabbit antibodies against Nrf2, TGF-β1 and Smad3 (1:1000) (ProteinTech Group, Inc., Chicago, IL, USA) and mouse monoclonal antibody against Lamin B1 (Abcam, Cambridge, UK) or β-actin (1:1000) (Santa Cruz Biotechnology, Santa Cruz, CA, USA) were employed. Peroxidase-conjugated goat anti-rabbit or anti-mouse IgG (1:5000) (Santa Cruz Biotechnology, Santa Cruz, CA, USA) were employed as the secondary antibodies. The specific protein bands were visualized using the enhanced western luminescent detection kit (Vigorous Biotechnology, Beijing, China). The results were quantified by densitometry using Image J software, and the densitometry results were normalized relative to the Lamin B1 or β-actin bands.

### 4.9. RNA Extraction and Real-Time Quantitative Polymerase Chain Reaction (PCR)

Total RNAs from liver tissues were isolated by using the TRIzol extraction kits according to the manufacturer’s instructions (Invitrogen Inc., Carlsbad, CA, USA). The quality of RNA was evaluated by the ratio of optical density (OD) at 260 nm and 280 nm, and all ratios of OD_260_/OD_280_ were in the range of 1.8~2.1. The production of cDNA was obtained from total RNA by using the prime scriptTM RT reagent kit (Takara Biotechnology, Co., Ltd., Dalian, China). RT-PCR was performed with a SYBR Green qPCR Kit (TaKaRa). The PCR conditions and primers used were as follows: *TGF-β1* forward: 5′-GAT TGT TGC CAT CAA CGA CC-3′; *TGF-β1* reverse: 5′-GTG CAG GAT GCA TTG CTG AC-3′ [5]; *Smad3* forward: 5′-CCA GCA CAC AAT AAC TTG GA-3′; *Smad3* reverse: 5′-AGA CAC ACT GGA ACA GCG GA-3′ [5]; *Nrf2* forward: 5′-CAC ATT CCC AAA CAA GAT GC-3′; *Nrf2* reverse 5′-TCT TTT TCC AGC GAG GAG AT-3 [56]′; *HO-1* forward: 5′-CGT GCT CGA ATG AAC ACT CT-3′; *HO-1* reverse: 5′-GGA AGC TGA GAG TGA GGA CC-3′ (GenBank accession No. NM_010442.2); *β-actin* forward, 5′-ATT CGT TGC CGG TCC ACA CCC-3′; *β-actin* reverse, 5′-GCT TTG CAC ATG CCG GAG CC-3′ (GenBank accession No. NC_034574.1). PCR was performed by using following reaction conditions: initial activation of Taq DNA polymerase at 95 °C for 5 min, 40 cycles of 30 s at 95 °C for denaturing, 30 s at 60 °C for annealing, and 30 s at 72 °C for elongation. The RT-PCR test was analyzed by ABI QuantStudio™7 detection system (Applied Biosystem, Foster City, CA, USA). After the amplification phase, a melting curve analysis was conducted to eliminate the possibility of non-specific amplification or primer dimer formation. All reactions were conducted in triplicate. *β-actin* was used as an internal control, and fold change in gene expression was calculated using the threshold cycle method (2^−ΔΔ*CT*^) [57].

### 4.10. Statistical Analysis

All data are expressed as the mean ± standard deviation (S.D.). Data from all the treatment groups were analyzed with one-way analysis of variance, followed by the LSD post hoc test using SPSS V13.0 (SPSS Inc., Chicago, IL, USA). Statistical significance was set at *p* < 0.05.

## Figures and Tables

**Table 1:** Effect of curcumin pre-treatment on the levels of oxidative-stress markers in the liver tissues of mice treated with CCl_4_.

Treatment Group						
Biomarker	Control	Cur 200	CCl_4_	Cur 50 + CCl_4_	Cur 100 + CCl_4_	Cur 200 + CCl_4_
MDA (mmol/mg protein)	1.84 ± 0.22	1.77 ± 0.25	2.79 ± 0.31 **	2.54 ± 0.37 ^#^	2.41 ± 0.18 ^#^	2.14 ± 0.39 ^##^
SOD (U/mg protein)	110.2 ± 24.20	116.4 ± 13.94	78.4 ± 10.5 **	84.6 ± 15.1	94.3 ± 12.1 ^##^	99.7 ± 17.2 ^##^
CAT (U/mgprotein)	130.7 ± 15.8	138.4 ± 21.4	86.5 ± 15.1 **	98.1 ± 10.6 ^#^	106.7 ± 20.1 ^#^	118.2 ± 17.8 ^##^
GSH (mmol/mg protein)	57.1 ± 6.7	62.6 ± 8.1	38.2 ± 9.4 **	43.3 ± 7.8	46.8 ± 10.3 ^#^	50.4 ± 9.6 ^#^

Results are presented as the mean ± S.D. (*n* = 8 in each group). Cur, curcumin; MDA, malondialdehyde; SOD, superoxide dismutase; CAT, catalase; GSH, glutathione. ** *p* < 0.01, compared to the control; ^#^ *p* < 0.05 and ^##^ *p* < 0.01 compared to the solely CCl_4_ treatment group.
